# Computertomographie des Thorax bei Kindern: Indikationen, technische Optimierung und Zukunftsperspektiven

**DOI:** 10.1007/s00117-025-01533-y

**Published:** 2025-11-10

**Authors:** Eszter Nagy, Erich Sorantin, Sebastian Tschauner

**Affiliations:** Klinische Abteilung für Kinderradiologie, Universitätsklinik für Radiologie, Auenbruggerplatz 34, 8036 Graz, Österreich

**Keywords:** Kinderradiologie, Strahlenschutz, ALARA, Hochauflösende Computertomographie, Photon-Counting-Computertomographie, Pediatric radiology, Radiation Protection, ALARA, High resolution computed tomography, Photon-counting computed tomography

## Abstract

**Hintergrund:**

Die Computertomographie (CT) des Thorax ist ein unverzichtbares Werkzeug in der pädiatrischen Radiologie, das jedoch besondere Herausforderungen mit sich bringt.

**Ziel der Arbeit:**

Dieser Übersichtsartikel stellt einen praxisorientierten Ansatz zur Optimierung der thorakalen CT bei Kindern vor, von der Indikationsstellung über die technische Durchführung bis hin zu neuen Technologien.

**Material und Methoden:**

Es erfolgte eine kritische Zusammenstellung relevanter wissenschaftlicher Ergebnisse und Leitlinien.

**Ergebnisse:**

Die Optimierung der Thorax-CT bei Kindern erfordert eine strenge Indikationsstellung, die Anpassung der Scanparameter (v. a. Röhrenspannung) an die kindliche Physiologie und den Einsatz spezifischer Protokolle (z. B. hochauflösende CT [HRCT], Low-dose-CT). Bildrekonstruktion und Post-Processing (Maximum-Intensitäts-Projektion [MIP], Minimum-Intensitäts-Projektion [MinIP], Volumen-Rendering-Technik [VRT]) sind für die Diagnostik entscheidend. Dosismonitoring mittels diagnostischer Referenzwerte (DRLs) und Achievable Doses (ADs) ist essenziell.

**Diskussion:**

Neuere Entwicklungen, insbesondere die Photon-Counting-CT, werden hinsichtlich ihres Potenzials zur Verbesserung der Bildqualität und Reduktion der Strahlenexposition im Kontext der thorakalen Bildgebung erörtert.

## Abstract (en)

**Background:**

Computed tomography (CT) of the chest is an indispensable tool in pediatric radiology, but it imposes particular challenges.

**Objective:**

This review article presents a practice-oriented approach to optimizing thoracic CT in children, from clinical indications to technical execution and new technologies.

**Materials and methods:**

A critical review of relevant scientific publications and guidelines was performed.

**Results:**

Optimizing pediatric chest CT requires strict indications, adaptation of scan parameters (especially tube voltage) to pediatric physiology, and the use of specific protocols (e.g., high resolution CT [HRCT], low-dose CT). Image reconstruction and postprocessing (maximum intensity projection [MIP], minimum intensity projection [MinIP], volume rendering technique [VRT]) are crucial for diagnosis. Dose monitoring using diagnostic reference levels (DRLs) and ADs is essential.w

**Conclusion:**

Recent developments, particularly photon-counting CT, are discussed regarding their potential to improve image quality and reduce radiation exposure in the context of thoracic imaging.

Die Computertomographie (CT) des Thorax bei Kindern ist ein Balanceakt zwischen maximaler diagnostischer Aussagekraft und dem Gebot des konsequenten Strahlenschutzes. Aufgrund der einzigartigen Anatomie und Physiologie von Kindern können Protokolle von Erwachsenen nicht einfach adaptiert werden; sie erfordern ein tiefes Verständnis der physikalischen Prinzipien und der klinischen Fragestellung. Dieser Artikel bietet einen praxisnahen Leitfaden, um diese Herausforderung im klinischen Alltag zu meistern.

Die thorakale CT ist integraler Bestandteil der diagnostischen Algorithmen bei diversen Erkrankungen des Thorax bei Kindern und Jugendlichen. Bei der Planung sind die speziellen anatomischen und physiologischen Unterschiede zu berücksichtigen, die umso ausgeprägter sind, je jünger ein Patient ist. Die rasche Veränderung der Körperzusammensetzung mit einem initial hohen Wasseranteil und erst spärlich ausgebildeten mediastinalen Fettlogen führt zu einem inhärent kontrastarmen Bild, was die Differenzierung von Gefäßen und Lymphknoten erschwert und oft den Einsatz von Kontrastmittel unumgänglich macht. Das schnelle Wachstum bedingt eine erhöhte Stoffwechselrate mit höheren Atem- und Herzfrequenzen. Die schnelle Zirkulation ist eine der wichtigsten Herausforderungen bei einer CT-Angiographie (CTA). Der initial hohe Wassergehalt und die spärlich ausgebildeten mediastinalen Fettlogen führen bei pädiatrischen Patienten zu einer verhältnismäßig größeren Menge an Kontrastmittel. Bei der Anwendung jodhaltiger Kontrastmittel sollte auch die Unreife der Nieren bei Neugeborenen und Säuglingen berücksichtigt werden. Dieser Artikel beleuchtet die State-of-the-Art-Anwendung der Thorax-CT bei Kindern. Ausgehend von einer kritischen Diskussion der klinischen Indikationen werden die technischen Grundlagen der Dosisoptimierung detailliert erläutert. Darauf aufbauend werden spezifische Protokolle für häufige Fragestellungen sowie die entscheidende Rolle der Bildrekonstruktion und des Post-Processing vorgestellt. Ein Überblick über moderne Dosismonitoring-Strategien sowie ein Ausblick auf zukunftsweisende Technologien wie die Photon-Counting-CT runden die Darstellung ab.

## Indikationen für die Thorax-CT

Die Indikation für eine Thorax-CT muss streng gestellt und gegen alternative, strahlenfreie Methoden wie die Sonographie und Magnetresonanztomographie (MRT) abgewogen werden. Dennoch gibt es klare klinische Szenarien, in denen die Thorax-CT im Kindesalter aufgrund ihrer unübertroffenen räumlichen und zeitlichen Auflösung sowie ihrer Robustheit unverzichtbar ist. Die Rechtfertigung der Untersuchung bildet den ersten und wichtigsten Schritt des Strahlenschutzes.

### Angeborene Fehlbildungen

Bei Verdacht auf komplexe angeborene Anomalien der Atemwege, des Lungenparenchyms oder der thorakalen Gefäße ist die CT-Angiographie (CTA) oft die entscheidende Untersuchung für die definitive Diagnose und die präoperative Planung. Oft wird sie bereits im Neugeborenen- und Säuglingsalter notwendig.

- Vaskuläre Anomalien: Klinische Symptome wie Stridor oder Dysphagie bei Säuglingen können durch eine Kompression der Trachea oder des Ösophagus aufgrund von Gefäßanomalien (sog. Gefäßringe) verursacht werden. Die CT-Angiographie (CTA) mit 3D-Rekonstruktionen (VRT) ist hier der Goldstandard, um die komplexe Anatomie eines doppelten Aortenbogens oder einer Lungenarterienschlinge („pulmonary artery sling“) darzustellen und den genauen Ort und das Ausmaß der Kompression zu visualisieren [25].
- Bronchopulmonale Malformationen: Bei einem Lungensequester kann die CTA hilfreich sein, um die aberrierende, systemarterielle Versorgung des sequestrierten Lungenareals zu identifizieren. Bei großen kongenitalen pulmonalen Atemwegsmalformationen (CPAM) kann die CT das Ausmaß, den Masseneffekt auf das Mediastinum und eine eventuelle Hybridläsion präzise darstellen [24].
- Tracheo- und Bronchomalazie: Goldstandard ist die Bronchoskopie. Die dynamische Exspirations-CT kann einen dynamischen Kollaps der zentralen Atemwege darstellen, empfiehlt sich aber aufgrund der Strahlenexposition bei Kindern nicht.
- Fehlbildungen der Atemwege: Varianten und Fehlbildungen der Atemwege (z. B. akzessorische Bronchien, Trachealstenosen) können mittels CT-Bildgebung und Nachverarbeitungsmethoden wie virtueller Bronchoskopie zuverlässig beurteilt werden.

### Infektiöse und entzündliche Erkrankungen

Bei komplizierten oder therapierefraktären Pneumonien kann die CT entscheidende Zusatzinformationen liefern, die über das konventionelle Röntgenbild oder die Sonographie hinausgehen. Die Indikation wird aber zunehmend durch die MRT ersetzt.

- Komplizierte Pneumonie: Bei Verdacht auf eine nekrotisierende Pneumonie ist die CT neben kontrastmittelgestütztem Ultraschall und der MRT eine Methode, die die typischen, nichtkontrastmittelaufnehmenden Areale und die Bildung von Pneumatozelen sicher nachweisen kann. Sie ist ebenfalls überlegen in der Detektion von Lungenabszessen und deren Beziehung zum Bronchialsystem sowie zur Differenzierung von Pleuraempyemen, die eine chirurgische Intervention erfordern könnten [3].
- Immungeschwächte Patienten: Bei Kindern unter Immunsuppression (z. B. nach Chemotherapie oder Transplantation) kann die CT spezifische Muster aufzeigen, die auf bestimmte opportunistische Erreger hinweisen. Beispiele sind das „Halo-Zeichen“ bei der angioinvasiven Aspergillose oder das Tree-in-bud-Muster bei einer infektiösen Bronchiolitis (z. B. durch Mykobakterien).

### Interstitielle Lungenerkrankungen

Die CT ist das wichtigste bildgebende Verfahren in der Diagnostik der vielfältigen und seltenen Gruppe der interstitiellen Lungenerkrankungen im Kindesalter. Die Bildgebung ist hier oft der Schlüssel zur Eingrenzung der breiten Differenzialdiagnose und dient der Erkennung und Charakterisierung von Mustern wie Milchglastrübungen, Mosaikperfusion, Retikulationen oder zystischen Veränderungen. Die hochauflösende CT (HRCT) besteht üblicherweise aus Inspirationsaufnahmen mit Exspirationsschichten. Bei Kleinkindern muss die Notwendigkeit zusätzlicher Exspirationsaufnahmen und des sich daraus ableitenden Atemstillstands (in Intubationsnarkose) streng überprüft werden. Spezifische Diagnosen können meist auch ohne Exspirationsaufnahmen vermutet werden, wie z. B. die geografische Verteilung von Milchglastrübungen bei der neuroendokrinen Hyperplasie des Säuglingsalters (NEHI; [14]). Darüber hinaus kann die CT entscheidend für die Wahl des optimalen Ortes für eine Lungenbiopsie sein.

### Kinderonkologie

In der pädiatrischen Onkologie ist die Thorax-CT für das initiale Staging und die Verlaufskontrolle unerlässlich. In diesem Kontext kann die Thorax-CT je nach lokalen Gegebenheiten auch im Rahmen eines nuklearmedizinischen Stagings mittels Positronen-Emissions-Tomographie (PET) erfolgen.

- Staging von Primärtumoren: Bei Tumoren des Mediastinums (z. B. Lymphom, Neuroblastom) oder der Thoraxwand (z. B. Ewing-Sarkom) ist eine kontrastverstärkte CT entscheidend, um die Tumorausdehnung, die Infiltration von Nachbarstrukturen und insbesondere die Ummauerung oder den Verschluss von Gefäßen („vessel encasement“) zu beurteilen. Diese Informationen sind entscheidend für die Therapieplanung und die Einschätzung der Resektabilität [47].
- Detektion von Lungenmetastasen: Die CT ist die sensitivste Methode zur Detektion pulmonaler Metastasen. Für Verlaufsuntersuchungen sind hier etablierte Low-dose-Protokolle der Standard, die eine adäquate diagnostische Sicherheit bei minimaler Strahlenexposition bieten. Die Verwendung von Maximum-Intensitäts-Projektions(MIP)-Rekonstruktionen verbessert die Detektionssicherheit zusätzlich. Eine Kontrastmittelgabe ist meist nicht notwendig.

### Trauma

Im Rahmen des Polytrauma-Managements bei Kindern ist die Thorax-CT (oft als Teil einer Ganzkörper-CT) dem konventionellen Röntgenbild in der Detektion relevanter Verletzungen weit überlegen. Sie identifiziert zuverlässig kleine oder okkulte Pneumo- und Hämatothoraces, Lungenkontusionen und -lazerationen sowie Frakturen. Von größter Wichtigkeit ist die CTA bei Verdacht auf eine Verletzung der großen Gefäße, wie einer traumatischen Aortendissektion. Hier können moderne Split-Bolus-Protokolle in einem einzigen Scan eine umfassende Beurteilung der arteriellen, venösen und parenchymatösen Strukturen ermöglichen und so Zeit und Dosis sparen [17].

## Technische Durchführung

Bei der Optimierung von CT-Protokollen geht es darum, bei geringstmöglicher Strahlenexposition eine diagnostische Bildqualität zu erreichen. Die diagnostische Bildqualität ist nicht gleichbedeutend mit perfekter Bildqualität und von der klinischen Fragestellung abhängig. Der wichtigste Deskriptor der Bildqualität ist das Bildrauschen. In den folgenden Abschnitten stellen wir einen praktischen Ansatz für die thorakale CT-Bildgebung bei Kindern vor. Wir heben die wichtigsten Faktoren hervor, die bei der Betrachtung der Strahlenexposition und der resultierenden Bildqualität zu berücksichtigen sind.

### Klinische Zuweisung

Jede Untersuchung beginnt mit einer klinischen Zuweisung, die die klinische Fragestellung und die zu scannende Körperregion enthält. Gemäß dem ALARA-Prinzip („as low as reasonably achievable“) und den daraus abgeleiteten Kampagnen von Image Gently [40] und EuroSafe Imaging [45] müssen Kinderradiologen im klinischen Entscheidungsprozess involviert werden, um die am besten geeignete Bildgebungsmodalität auszuwählen, welche die Frage der überweisenden Ärzte bei geringstmöglicher Strahlenexposition beantwortet.

Standards der Europäischen Union und internationale Leitlinien können ebenfalls die klinische Entscheidungsfindung leiten [2]. Es ist zu beachten, dass klinisch-radiologische Entscheidungen immer auf individueller Basis getroffen werden sollten. In vielen Fällen ist durch alternative Bildgebungsmöglichkeiten eine CT-Untersuchung vermeidbar, und die CT-Protokolle sind an individuelle klinische Fragestellungen anpassbar. Praktisch sollten Körpergewicht, Größe und Kreatinin und geschätzte glomeruläre Filtrationsrate (eGFR) vermerkt werden. Letztere sind obligatorisch, wenn ein intravenöses Kontrastmittel erforderlich ist. Darüber hinaus sollten Informationen über Komorbiditäten (z. B. Schilddrüsenerkrankungen) entweder in der Überweisung enthalten sein oder mit dem Pädiater besprochen werden. Ein aktueller TSH-Wert sollte verlangt werden.

### Patientenpositionierung

Die medizinisch-technischen Radiologieassistenten bzw. -assistentinnen (MTRA) sind für die korrekte Positionierung des Patienten im Isozentrum der Gantry verantwortlich. Ein nichtzentrierter Scan in Bezug zur Gantrymitte (oben – unten und seitlich) führt zu einer Fehlausrichtung gegenüber dem Bowtie-Filter, was sich negativ auf die Strahlenverteilung auswirkt und zu Expositionsunterschieden von bis zu 50 % im Vergleich zur korrekten zentralen Positionierung führen kann [42]. Wenn der Patient näher an die Röntgenröhre bewegt wird, erscheint seine Silhouette größer, was aufgrund der automatischen Belichtungssteuerung (AEC) zu einer Erhöhung der Strahlenexposition führen kann. Liegt der Patient hingegen weiter von der Röhre entfernt, erscheint seine Silhouette kleiner und die AEC regelt die Strahlenexposition herunter, wodurch die Bildqualität durch erhöhtes Rauschen verschlechtert wird. Mittlerweile sind auf künstlicher Intelligenz (KI) basierende Systeme verfügbar, um eine korrekte zentrale Positionierung sicherzustellen [4].

Zusätzlich zur Zentrierung beeinflusst auch die Position der Arme die Strahlenexposition. Idealerweise sollte der Patient bei Thoraxuntersuchungen mit den Händen über dem Kopf untersucht werden. Wenn der Patient dazu nicht bereit oder in der Lage ist (z. B. bei einer Fraktur der oberen Extremität), ist die beste Lösung, die Hände mit gestreckten Armen vor dem Körper zu kreuzen und einige Handtücher zwischen Arme und Rumpf zu legen. Mit diesem Ansatz wird der anteroposteriore (a.-p.) Durchmesser vergrößert und die Patientengeometrie abgerundeter, was zu einer homogeneren Strahlenverteilung und weniger Aufhärtungsartefakten durch die Humeri führt. Die Physik dahinter wird durch die Brooks-Formel erklärt, die den Zusammenhang zwischen Strahlenexposition und Bildrauschen beschreibt [6]. Gemäß dieser Formel muss bei einer Zunahme des Patientendurchmessers um 4 cm (z. B. wenn die Hand seitlich am Körper liegt) die Strahlenexposition verdoppelt werden, um die Bildqualität zu erhalten.

### Abschirmung (Shielding)

Die Patientenabschirmung in der CT-Untersuchung ist ein kontroverses Thema, über das in der Vergangenheit viele Diskussionen stattgefunden haben. Um diese Probleme zu überwinden, wurde eine europäische Empfehlung veröffentlicht [15], die von der Strahlenschutzkommission (SSK) weitgehend übernommen wurde [39]. Gemäß diesem Konsensusdokument ist mit moderner CT-Technologie eine In-Plane- oder Out-of-Plane-Abschirmung bei den meisten Fällen nicht notwendig.

### Topogramm

Der nächste Schritt in der Bildgebungskette ist das Scout-Bild (Topogramm). In der Regel werden zwei Scout-Bilder (a.-p. oder p.-a. und lateral) erstellt, um die korrekte Positionierung des Patienten im Isozentrum der Gantry sicherzustellen. Dabei wird die p.-a.-Ansicht der a.-p.-Ansicht vorgezogen, da dies eine signifikante Reduktion der Strahlenexposition strahlenempfindlicher Organe wie Brust und Schilddrüse bedeutet [38]. Allerdings kann die Gesamtstrahlenexposition der Scout-Ansicht mit der p.-a.-Ansicht zunehmen, da Strukturen hoher Dichte (Rippen, Wirbel) näher an der Röhre liegen [28]. Bemerkenswert ist, dass bei jüngeren Patienten in diesen Bereichen weniger rotes Knochenmark vorhanden ist, wodurch die regionale Strahlenempfindlichkeit abnimmt [1]. Um diesen möglichen Anstieg der Strahlenexposition auszugleichen, ist eine sorgfältige Anpassung der Belichtungsparameter an die Größe des Patienten erforderlich. Die Länge von Scout und Scan sollte an die klinische Fragestellung angepasst werden, um ein Überscannen zu vermeiden. Bei Niedrigdosis-CT-Scans machen Scout-Bilder zunehmend einen signifikanten Anteil (bis zu einem Fünftel) der gesamten Strahlendosis der Studie aus, weshalb die Expositionsparameter des Scouts für Kinder optimiert werden sollten [44].

### Scantyp

Die pädiatrische Computertomographie (CT) wird hauptsächlich im Spiral- (auch: helikal) oder Volumen-Scanmodus (auch: axial) durchgeführt. Grundsätzlich wird immer versucht, mit einer Serie/Belichtung ohne Tischvorschub auszukommen, was insbesondere bei Neugeborenen oft ausreichend und „strahlensparend“ ist. Nur bei entsprechenden klinischen Fragestellungen, wie beispielsweise Gefäßdarstellungen, Tumoren oder Infektionen, werden Zusatzserien oder Kontrastmittelphasen angefertigt. Während die Spiral-CT durch den Over-Ranging- und den Over-Beaming-Effekt belastet ist, spielt der Over-Beaming-Effekt bei der Volumen-CT durch den fehlenden Tischvorschub nur bei mehreren überlappenden Rotationen eine nennenswerte Rolle.

Als Over-Ranging wird eine Erweiterung des Scanbereichs um zumindest eine halbe Gantry-Rotation bezeichnet, die für die Rekonstruktion der jeweils letzten Schichten entlang der Z‑Achse eines Patienten erforderlich ist. Over-Ranging ist direkt proportional zur Kollimationsbreite und zum Pitch. Da Over-Ranging unabhängig von der Scanlänge ist, ist die relative Strahlenexposition durch Over-Ranging höher, wenn die Scanlänge kurz ist (z. B. pädiatrischer Thorax; [11]). Einige herstellerspezifische Lösungen, wie dynamische Kollimation oder hybride Rekonstruktionsalgorithmen, wurden eigens entwickelt, um die Strahlenexposition durch Over-Ranging abzuschwächen [35]. Trotzdem sollte nach Möglichkeit der axiale Scanmodus bevorzugt werden.

Der Over-Beaming-Effekt bezeichnet eine redundante Strahlenexposition pro Rotation, die umgekehrt proportional zur Anzahl der Detektorzeilen ist. Daher sollte bei Kindern ein Mehrzeilen-CT mit breiten Detektoren (z. B. 16 cm) verwendet werden, da der Over-Beaming-Effekt bei diesen Geräten nicht signifikant ist.

Aufgrund der Detektorkonfiguration neuerer CT-Scanner ist der Einzelschichtmodus nicht immer verfügbar, und es müssen geräteabhängig kleine Volumina gescannt werden. Daher kann dieser Scanmodus als veraltet angesehen werden.

Moderne Single-Source- und Dual-Source-CT(DSCT)-Scanner bieten auch einen Dual-Energy-Scanmodus an. Die Erfahrungen bei pädiatrischen Patienten sind noch begrenzt, allerdings weisen Studien darauf hin, dass DSCT Kinder vergleichbaren oder sogar geringeren Strahlenexpositionen aussetzt als Single-Energy-CT [37]. Nach unserer Erfahrung erfordert das Single-Source-Dual-Energy-Scannen etwa 30 % mehr Strahlenexposition als der Single-Energy-Modus, was diesen Scanmodus für kleine Patienten eher unattraktiv macht. Die einzige Indikation für Dual-Energy-Single-Source-Scans in unserer Institution ist die Metallartefaktreduktion bei der Extremitäten-CT [20].

### Spezifika der Lungen-CT

Der Begriff der hochauflösenden CT (HRCT) der Lungen hat sich im Laufe der Zeit gewandelt. Während er historisch eine spezielle Technik mit dünnen Schichten und scharfem Rekonstruktionsalgorithmus, also einem *harten* Kernel, beschrieb, sind diese Parameter heute dank moderner Multidetektor-Scanner Standard. Jeder volumetrische Thorax-CT-Datensatz wird routinemäßig mit einem hochauflösenden Lungenkernel rekonstruiert. Dennoch hat der Begriff „HRCT“ eine klinische Relevanz als spezifischer Protokollauftrag behalten. Er signalisiert die Notwendigkeit, die Untersuchung gezielt auf die Diagnostik des Lungenparenchyms und der kleinen Atemwege auszurichten. Ein modernes HRCT-Protokoll beinhaltet daher oft nicht nur die Akquisition in tiefer Inspiration, sondern obligat auch Serien in Exspiration zur Detektion eines Air-Trappings sowie optional Scans in Bauchlage zur Differenzierung interstitieller Veränderungen von lageabhängigen Atelektasen.

#### Serie in Inspiration.

Die Basis bildet eine volumetrische Akquisition in tiefer, gehaltener Inspiration. Diese wird mit einer für die Diagnostik ausreichenden, aber optimierten Dosis gefahren. Die Rekonstruktion erfolgt mit einem kantenschärfenden Algorithmus (High-Spatial-Frequency-Kernel), der zwar das Bildrauschen erhöht, aber die Definition kleinster Strukturen wie Bronchialwände und Interlobulärsepten maximiert. Die Schichtdicke sollte bei 1,0 mm oder weniger liegen.

#### Exspirationsserie.

Dies ist ein obligater Bestandteil bei Verdacht auf eine Erkrankung der kleinen Atemwege. Die Sequenz wird am Ende einer forcierten Exspiration akquiriert. Das Ziel ist die Detektion von Air-Trapping, also Arealen, die sich in der Ausatmung nicht erwartungsgemäß entlüften und daher im Vergleich zum umgebenden, normal kollabierenden Parenchym eine niedrigere Dichte (dunkler) aufweisen. Dies ist ein Schlüsselbefund bei Erkrankungen wie der Bronchiolitis. Die Exspirationssequenz kann und soll als Low-dose-Scan durchgeführt werden, da hier primär Dichteunterschiede und nicht feinste morphologische Details beurteilt werden.

#### Serie in Bauchlage („Prone Scan“).

Bei Nachweis von Milchglastrübungen oder feinen retikulären Veränderungen in den dorsalen, basalen Lungenabschnitten ist eine kurze Sequenz in Bauchlage oft diagnostisch entscheidend. Sie dient der Differenzierung zwischen echten, pathologischen Veränderungen (z. B. frühe Fibrose) und reversiblen, schwerkraftbedingten Kompressionsatelektasen („dependent atelectasis“). Während Atelektasen in Bauchlage verschwinden und sich das Parenchym normal belüftet, persistieren fibrotische Veränderungen. Auch dieser Scan wird als Low-dose-Sequenz durchgeführt.

#### Serien in aufrechter Position.

**Abb. 1:**
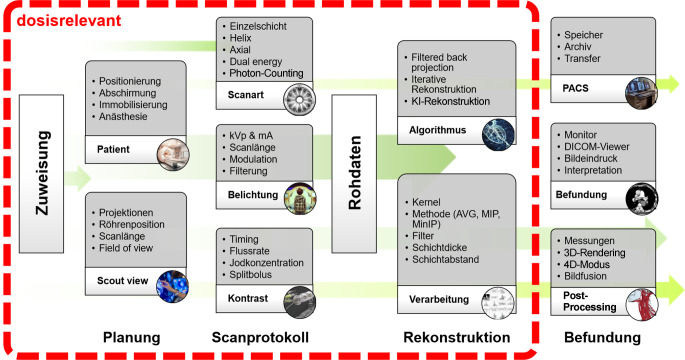
Schematische Darstellung der Abfolgeschritte zur Durchführung einer CT-Untersuchung im Sinne einer CT-Bildkette. Es muss beachtet werden, dass dosisrelevante Aspekte bereits mit der Zuweisung zu einer Untersuchung beginnen und sich über die Planung und Auswahl des Scanprotokolls bis zum Schritt der Bildrekonstruktion weiterziehen. *AVG* Mittelwert, *DICOM* Digital Imaging and Communications in Medicine, *kVp* Spitzenkilovoltage, *mA* Milliampere, *MinIP* Minimum-Intensitäts-Projektion, *MIP* Maximum-Intensitäts-Projektion, *PACS* Picture Archiving and Communication System. (Adaptiert nach [31])

Eine neuartige, wenngleich noch wenig verbreitete Methode zur funktionellen Lungenbildgebung ist die CT in aufrechter, sitzender oder stehender Position. Diese wird mit speziellen Area-Detektor-CT (ADCT) oder Cone-Beam-CT Systemen durchgeführt. Der diagnostische Mehrwert kann erhöht sein, da die Methode die Lunge unter physiologischer Schwerkraftbelastung und in ihrer funktionellen Atemruhelage abbildet. Im Gegensatz zur liegenden Position werden die dorsalen Lungenabschnitte nicht komprimiert, was eine Beurteilung ohne den Störfaktor der „dependent atelectasis“ von vornherein ermöglicht ([18]; Abb. 1).

### Belichtungseinstellungen

Die Beziehung zwischen Röhrenstrom (in mA) und Strahlenexposition ist linear, sodass eine verdoppelte mA zu einer doppelt so hohen Strahlenexposition führt (unter der Annahme, dass alle anderen Faktoren konstant sind). Mit Erhöhung der mA wird das Bildrauschen verringert und damit die Bildqualität gesteigert, während der Bildkontrast nicht beeinflusst wird.

**Abb. 2:**
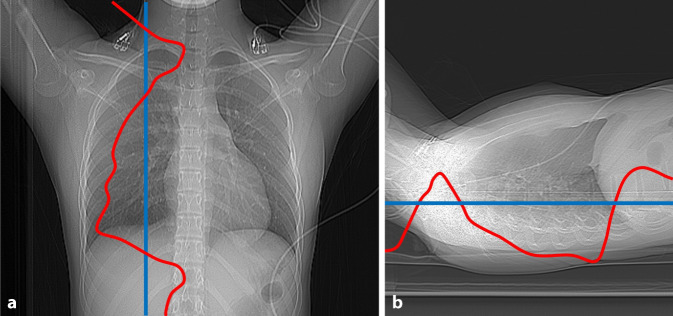
Topogramme in p.-a. (**a**) und lateraler (**b**) Projektion. Die *rote Linie* markiert das AEC-Dosisprofil. Die *blaue Linie* stellt eine zu hoch eingestellten minimalen mAs-Wert der AEC dar, der die Exposition nach unten entlang der roten Modulationslinie bei zu hoher Einstellung limitieren könnte. (Mod. nach [38])

Automatische Röhrenstrommudulationssysteme („automatic exposure control“, AEC) werden üblicherweise zur Reduzierung der Strahlenexposition empfohlen [8]. AEC-Systeme bewerten das Röntgenstrahlen-Abschwächungsprofil des Körpers im Scanbereich basierend auf der Scout-Ansicht und passen den Röhrenstrom an, um die Bildqualität während des gesamten Scans aufrechtzuerhalten. Die verschiedenen AEC-Systeme definieren die Bildqualität auf unterschiedliche Weise: „Bildrauschen“ und „Rauschindex“ werden von Canon (ehemals Toshiba) bzw. GE Healthcare verwendet, während Siemens den Begriff „Referenz-mAs“ nutzt [28]. Der Bediener wählt die minimalen und maximalen mAs-Werte für die AEC aus. Um die bestmögliche Reduktion der Strahlenexposition zu erreichen, ist es ratsam, den Minimalwert auf den niedrigsten Wert einzustellen (Abb. 2).

Die Röhrenspannung (kV) hat ebenfalls einen starken Einfluss auf die Strahlendosis. Die Beziehung zwischen kV und Strahlenexposition ist quadratisch. Das bedeutet eine 50 %ige Strahlenreduktion, wenn die Röhrenspannung von 120 kV auf 80 kV gesenkt wird und alle anderen dosisrelevanten Parameter konstant gehalten werden. Andererseits führt die reduzierte kV zu größeren Abschwächungsunterschieden in den Geweben, die auf den photoelektrischen Effekt zurückzuführen sind. Dadurch erhöht sich der intrinsische Kontrast. [48]. Dieses Phänomen reduziert auch das erforderliche Volumen des zu applizierenden Kontrastmittels [32]. Die Bildgebung mit reduzierter Röhrenspannung kommt jedoch nur kleinen Patienten zugute. Je größer die Patienten sind, desto stärker werden die Bilder durch Aufhärtungsartefakte belastet, wodurch sich die diagnostische Bildqualität verschlechtert. Leider gibt es keine klare Grenze bezüglich der Patientengröße und des Ausmaßes der kV-Reduktion. Dies kann von Scanner zu Scanner variieren.

Wie bereits erwähnt, bedeuten die niedrigeren kV-Werte einen erhöhten inhärenten Gewebekontrast und führen zu einer Verschiebung zu höheren Hounsfield-Einheiten. Eine Diskrepanz zwischen den Röhrenspannungen des Bolus-Tracking-Monitoring-Scans und der folgenden Angiographie kann aufgrund des veränderten Gewebekontrasts zu einem verspäteten Scanstart führen. Daher muss die Röhrenspannung für das Bolus-Tracking und für die diagnostische Angiographie gleich sein, und der Bolus-Tracking-Schwellenwert muss an die geänderte Röhrenspannung angepasst werden [41].

Gemäß der Brooks-Formel ist die Schichtdicke umgekehrt proportional zur Strahlenexposition. Eine halbe Schichtdicke erfordert somit die doppelte Strahlenexposition für das gleiche Bildrauschen, welches die Bildqualität maßgeblich definiert [38]. Um einen Partialvolumeneffekt zu vermeiden, sollte das Inkrement 50 % betragen.

Der Pitch ist definiert als Tischvorschub pro Rotation, geteilt durch die Kollimation. Ein höherer Pitch führt zu einem schnelleren Scan und folglich zu weniger Bewegungsartefakten, was eine Vollnarkose bei DSCT vermeiden könnte [22, 41]. Bemerkenswerterweise kann der Scanner bei erhöhtem Pitch den Röhrenstrom automatisch erhöhen, um eine Beeinträchtigung der Bildqualität zu vermeiden, und der Over-Ranging-Effekt kann zunehmen [27]. Daher wird empfohlen, einen Pitch von mehr als 1,5 bei Single-Source-CT zu vermeiden und den Pitch bei DSCT auf maximal 3,4 zu begrenzen. Moderne CT-Scanner wählen den optimalen Pitch automatisch anhand aller anderen Scanparameter aus, wodurch der Bedienereingriff minimiert wird.

### Zusätzliche Röhrenfilterung

Röntgenröhren emittieren ein Spektrum unterschiedlicher Röhrenspannungen bis zur Spitzenkilovoltage (kVp). In den meisten Fällen werden niedrig energetische Spektren als nicht nützlich für die Bildgebung angesehen. Tatsächlich werden niedrige Kilovoltagen typischerweise im Körper gestreut und absorbiert, ohne den Detektor je zu erreichen. In kontrastreichen Szenarien (z. B. Luft zu Weichteil oder Weichteil zu Knochen) hat sich eine zusätzliche Zinnfilterung zur Reduzierung der Strahlenexposition als wirksam erwiesen. Diese Funktion ist jedoch nur bei einer begrenzten Anzahl von Scannern verfügbar.

### Kontrastmittel

Bei der Gabe von Kontrastmitteln in der CT sind zusätzliche Überlegungen zur Strahlenbelastung des Patienten erforderlich. Ein Bolus-Tracking wird bei pädiatrischen Patienten üblicherweise für die Angiographie empfohlen. Da sich der Bolus-Tracking-Monitoring-Scan bei der Thorax-CT-Angiographie normalerweise im Bereich der strahlenempfindlichen Brustdrüsen befindet, ist eine Reduktion der Strahlenexposition angezeigt, falls möglich. Hinsichtlich der Belichtungseinstellungen sollte die Röhrenspannung an den diagnostischen Scan angepasst werden. Der Röhrenstrom kann jedoch reduziert werden, wodurch sich das Bildrauschen bei unverändertem Bildkontrast erhöht, was für das Bolus-Tracking entscheidend ist. Basierend auf der Brooks-Formel helfen die erhöhte Kollimation und Schichtdicke, die Strahlenexposition und das Rauschen zu reduzieren. Je nach Ort der Kontrastmittelgabe können unterschiedliche Längen des Bolus-Trackings erforderlich sein. Um die potenziell erhöhte Strahlung zu reduzieren, sollte ein späterer Start des Bolustrackings oder die Reduzierung der Anzahl der Bolustracking-Bilder in Betracht gezogen werden. Bei kleinen Kindern mit beschleunigter Zirkulation bergen diese Ansätze jedoch das Risiko einer suboptimalen Kontrastanreicherung.

Mehrphasenprotokolle führen naturgemäß zu einer höheren Strahlenbelastung, sie sind in der Thorax-CT selten indiziert. In einigen klinischen Fällen (z. B. Polytrauma) können Bilder in mehreren Phasen mit einem Split-Bolus-Protokoll sichergestellt werden, was zu einer geringeren Strahlenexposition führt [17].

Während der Applikation des Röntgenkontrastmittels sollte darauf Acht gegeben werden, dass die Jodkonzentration auf das jeweilige Alter und die Untersuchungsart angepasst wird. Dies kann entweder über die Verwendung unterschiedlicher Kontrastmittelvarianten oder -produkte erfolgen, oder durch entsprechende Verdünnung des Kontrastmittels. Stark konzentriertes Kontrastmittel kann den Röntgenstrahl so stark schwächen, dass daraus Streifenartefakte resultieren. Eine generelle Empfehlung zur etwaigen Verdünnung des Kontrastmittels ist nicht möglich, da mehrere Faktoren wie Kontrastmittelfabrikat, Jodkonzentration, Patientenalter, intravenöser Zugang und notwendiger Kontrastmittelfluss in die Gleichung einfließen. Verdünnungen von Kontrastmittel mit beispielsweise 2 Teilen Kochsalzlösung und 1 Teil 350 mg Jod/ml sind in der Neonatalperiode nicht unüblich. Die CT-Protokolle werden bei der Verabreichung von intravenösem jodhaltigem Kontrastmittel häufig angepasst und die Röhrenspannung auf z. B. 80 kV reduziert. Dies erhöht den Effekt des Kontrastmittels und hilft dabei, Kontrastmittel- und Strahlendosen niedrig zu halten.

Strahleninduzierte DNA-Brüche sind eine neu beschriebene Nebenwirkung bei der Verwendung von jodhaltigen Kontrastmitteln. Die Anwendung eines jodhaltigen Kontrastmittels erhöht die Häufigkeit potenziell schädlicher DNA-Brüche, die direkt proportional zum injizierten Volumen ist [7, 10]. Die klinische Relevanz dieses Phänomens ist jedoch bis dato unbekannt.

## Bildrekonstruktion und Post-Processing

Die iterative Rekonstruktion ist ein wertvolles Werkzeug zur Reduzierung der Strahlendosis [29]. Die anspruchsvollere modellbasierte iterative Rekonstruktion ist jedoch zeitaufwändig, und daher ist ihre klinische Anwendung auf neuere CT-Scanner beschränkt [30]. Kürzlich wurde die auf Deep Learning (DL) basierende Bildrekonstruktion eingeführt. Bei dieser Technik werden neuronale Netze darauf trainiert, niedrig dosierte Rohdaten zu entrauschen. Dadurch verbessert sich die Bildqualität bei gleichzeitiger Dosisreduktion [5].

Die Interpretation von CT-Datensätzen des Thorax, insbesondere bei der Suche nach kleinen oder subtilen Pathologien, profitiert enorm von spezifischen Rekonstruktionsalgorithmen, die über die Standard-Axialbilder hinausgehen. Multiplanare Reformationen (MPR) in Weichteil- und Lungenkernel in allen 3 Raumrichtungen (axial, koronal, sagittal) können als Standardtechniken angesehen werden und sollten bei jedem Scan durchgeführt und archiviert werden. Darüber hinaus sind die wichtigsten Techniken die MIP, MinIP und die VRT. Auch sie kommen je nach Fragestellung routinemäßig zum Einsatz.

**Abb. 3:**
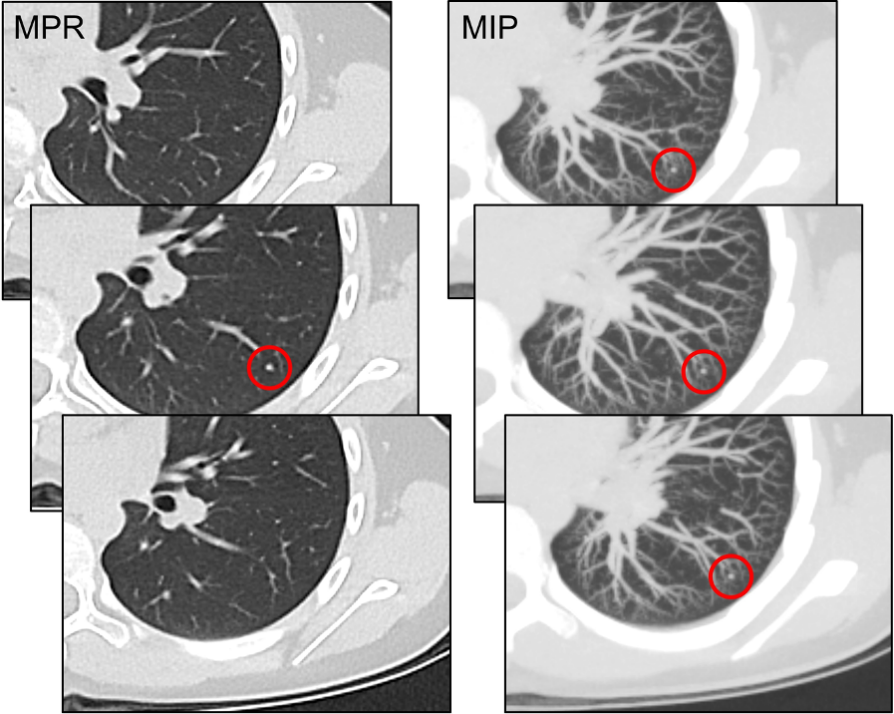
Gegenüberstellung zwischen multiplanarer Reformation (MPR, *links*) und Maximum-Intensitäts-Projektion (MIP, *rechts*) in der Darstellung eines kleinen Lungenrundherdes bei einer 10-jährigen kinderonkologischen Patientin. Während der punktförmige Herd in der MPR nur in einer Schicht klar sichtbar ist, so projiziert er sich in der MIP in mehrere Schichten, sodass er bei der dynamischen Durchsicht der Bilder einfacher erkennbar wird

Die Maximum-Intensitäts-Projektion rekonstruiert innerhalb eines definierten Volumens (Slab) nur die Voxel mit der höchsten Dichte (Hounsfield-Einheit) auf die Betrachtungsebene. Dies führt dazu, dass dichte Strukturen wie Lungennoduli und Gefäße hervorgehoben werden, während das wenig dichte Lungenparenchym unterdrückt wird. Die Hauptanwendung der MIP ist die verbesserte Detektion von pulmonalen Noduli, insbesondere bei onkologischen Staging- oder Follow-up-Untersuchungen. Studien haben wiederholt gezeigt, dass die zusätzliche Analyse von MIP-Bildern die Sensitivität für die Herddetektion im Vergleich zur alleinigen Betrachtung dünner Schichten signifikant erhöht. Die Noduli leuchten förmlich auf und heben sich vom komplexen Hintergrund der sich überlagernden Gefäße ab [13]. Eine rekonstruierte Schichtdicke von 10 mm hat sich als optimaler Kompromiss erwiesen, wobei für subsolide Herde eine Schichtdicke von 3 mm vorteilhaft ist [26]. Ist die Schicht zu dünn, ist der Effekt der Volumenprojektion gering. Ist sie zu dick, überlagern zu viele Gefäße die Noduli, was die Detektion wieder erschwert. Entscheidend ist ein stark überlappender Schichtabstand, z. B. von 10 mm Schichten. Damit wird verhindert, dass ein Herdbefund durch partielle Volumeneffekte am Rand einer Schicht *verloren* geht [21]. Eine MIP sollte niemals allein, sondern immer in Verbindung mit den Axialbildern befundet werden. Sie ist exzellent für die Detektion, aber schlecht für die Charakterisierung. Ein Beispiel findet sich in Abb. 3. Interne Eigenschaften eines Rundherdes (z. B. Fett, Verkalkungen, Milchglasanteile) gehen in der Projektion verloren. Zudem können sich kreuzende Gefäße oder Narben als Noduli maskieren und als falsch-positive Befunde in Erscheinung treten.

Die Minimum-Intensitäts-Projektion ist das Gegenteil der MIP, indem sie die Voxel mit der niedrigsten Dichte projiziert. Dadurch werden lufthaltige Strukturen wie die Atemwege oder pathologische Lungenareale mit verminderter Dichte hervorgehoben. Die MinIP ist das Werkzeug der Wahl zur Beurteilung von Erkrankungen, die mit einer verminderten Lungendichte oder pathologisch erweiterten, lufthaltigen Strukturen einhergehen. Dazu gehören beispielsweise:

- *Air-Trapping:* Bei der Beurteilung von Exspirationsscans ist die MinIP unübertroffen, um die typischen, scharf begrenzten dunklen Areale des Air-Trappings zu visualisieren [23].
- *Bronchiektasen und zystische Lungenerkrankungen:* Die Kontinuität und der Kaliberverlauf von pathologisch erweiterten Bronchien (z. B. bei zystischer Fibrose) lassen sich in einer MinIP oft über eine längere Strecke verfolgen als im einzelnen Axialbild.
- *Emphysem:* Zentrilobuläre oder panlobuläre Emphysemareale werden in der MinIP als klar definierte Dichteminderungen sichtbar. Diese Anwendung ist in der pädiatrischen Radiologie weniger relevant.

Ähnlich wie bei der MIP sind bei der MinIP Sliding thin-slab-Rekonstruktionen mit einer rekonstruierten Schichtdicke von 10 mm und mehr sowie hoher Überlappung sinnvoll.

**Abb. 4:**
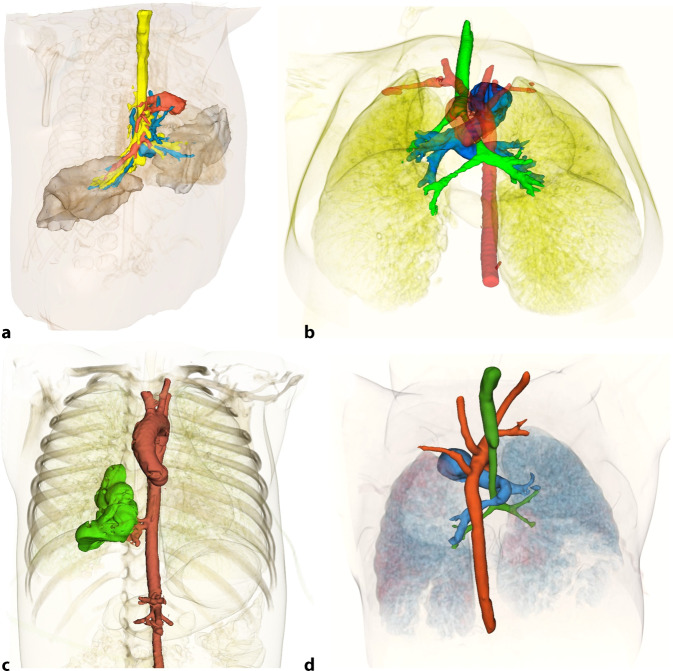
Beispiele für individualisierte Rekonstruktionen in Volumen-Rendering-Technik (VRT) zur präoperativen Planung. **a** Komplexe raumfordernde kongenitale pulmonale Atemwegsmalformation (CPAM) im Oberlappen rechts mit ausgeprägter Kompression des Mittel- und Unterlappens rechts, welche hier selektiv dargestellt werden. **b** Pulmonalisschlinge und sog. „bridging bronchus“ von links nach rechts. **c** Sequester im rechten Unterlappen. **d** Trachealstenose und Pulmonalisschlinge

Die Volumen-Rendering-Technik ist eine Nachverarbeitungsmethode, die aus dem gesamten volumetrischen CT-Datensatz dreidimensionale und in der Regel farbkodierte Modelle erstellt. Ihre unschätzbare Stärke liegt in der Fähigkeit, komplexe räumliche Beziehungen zwischen verschiedenen anatomischen Strukturen darzustellen, die mit rein zweidimensionalen Ansichten nur schwer zu erfassen wären. In Hinblick auf die pädiatrische Thorax-CT ist VRT wichtiger Bestandteil der Nachverarbeitung, indem sie Atemwege als „virtuelle Bronchoskopie“ darstellt und das Ausmaß von Stenosen oder einer Tracheomalazie eindrücklich visualisiert. Darüber hinaus findet die VRT vor allem in der präoperativen Planung Anwendung. Sie ist in der Lage, die Anatomie von Gefäßringen oder -schlingen und deren Kompression auf Organe wie Trachea und Ösophagus zu demonstrieren. Für Kinderchirurgen und Herzchirurgen sind diese 3D-Modelle eine essenzielle präoperative „Roadmap“, die die Planung komplexer Eingriffe erheblich verbessert. Darüber hinaus dient die VRT als exzellentes Kommunikationsinstrument, um dem interdisziplinären Team aus Pädiatern und Chirurgen komplexe Befunde verständlich zu vermitteln. Die VRT-Visualisierung erfolgt normalerweise relativ automatisiert anhand typischer HU-Schwellenwerte. Insbesondere bei jüngeren Patienten kann es aber notwendig sein, händisch die einzelnen Strukturen von Interesse zu segmentieren (vergleiche Abb. 4).

## Dosismonitoring und Protokolloptimierung

### CT-Dosisindizes als Grundlage des Monitorings

Mehrere Dosisindizes stehen zur Verfügung, um Strahlenexpositionsvergleiche zwischen verschiedenen Geräten und Patienten zu ermöglichen. Nach unserer Erfahrung werden diese wichtigen Indikatoren von Radiologen nicht ausreichend berücksichtigt. Wir erinnern daher im folgenden Abschnitt an die wichtigsten Parameter.

Der Volumen-CT-Dosisindex (CTDIvol) wird als standardisiertes Maß für die Strahlendosisleistung eines CT-Scanners verwendet. CTDI bezieht sich immer auf ein zylindrisches Referenzphantom mit 16 cm Durchmesser (Kopf) und 32 cm Durchmesser (Körper). CTDIvol repräsentiert die durchschnittliche absorbierte Dosis einer einzelnen Schicht und wird in mGy angegeben. CTDIvol ist ein berechneter Wert, der auf CTDIw basiert, einem gewichteten Durchschnitt des gemessenen CTDI über das Sichtfeld wie folgt: ein Drittel CTDI im Zentrum und zwei Drittel CTDI am Rand des zylindrischen Phantoms. Da kleine Kinder meist im Zentrum des Tisches liegen, sind CTDI-Werte bei pädiatrischen Patienten fehleranfällig. CTDIvol ermöglicht jedoch einen guten Vergleich zwischen CT-Scannern und CT-Protokollen.

Um die fehlende Berücksichtigung der Patientenmorphometrie im CTDI auf die resultierende Strahlendosis zu überwinden, wurde die größenspezifische Dosisabschätzung (SSDE) eingeführt. Sie ist die bestmögliche Annäherung an die vom Patienten absorbierte Dosis in der klinischen Praxis. SSDE korrigiert CTDIvol-Werte durch Multiplikation mit einem spezifischen Umrechnungsfaktor in Abhängigkeit vom effektiven Durchmesser des Patienten. Der effektive Patientendurchmesser wird an der größten Ausdehnung der gescannten Region gemessen. Ein bekannter Nachteil dieses Ansatzes ist, dass die unterschiedlichen Abschwächungsprofile von Thorax- und Abdominalregionen nicht routinemäßig in die Berechnung einbezogen werden [34].

Das Dosis-Längen-Produkt (DLP) ist ein Maß für die Strahlenleistung der CT-Röhre, das die Länge des CT-Scans und die Strahlenleistung entlang der z‑Achse berücksichtigt. DLP wird wie folgt berechnet: CTDIvol × Länge (cm). Je länger der Scan, desto höher die Strahlendosis. Mehrere Scans erhöhen ebenfalls das DLP. Da die DLP-Berechnung auf CTDIvol basiert, ignoriert sie naturgemäß die geometrischen Abmessungen des Patienten und beschreibt daher nicht die absorbierte Dosis. Es wurden jedoch k‑Faktoren veröffentlicht, die von der effektiven Körpergröße abhängen und die Umrechnung von DLP in effektive Dosis ermöglichen [33].

Effektive Dosen dienen als Maß für das Strahlenrisiko. Ihre Einheit ist Millisievert (mSv). Die Berechnung basiert auf körperregionsabhängigen Korrekturfaktoren. Heute sind für CT-Untersuchungen des Thorax in den meisten Fällen effektive Strahlendosen deutlich unter 1 mSv erzielbar.

### DRLs und ADs als Benchmarks für die Optimierung

Ein fundamentales Instrument des Dosismanagements ist der regelmäßige Abgleich der institutionellen Dosiswerte mit diagnostischen Referenzwerten (DRLs). DRLs sind definierte statistische Schwellenwerte, typischerweise das 75. Perzentil (3. Quartil) der Dosisverteilungen, die aus einer breiten Stichprobe von Untersuchungen an standardisierten Patientengruppen gewonnen werden. Sie fungieren nicht als Dosisgrenzwerte, sondern als wichtige Benchmarks: Eine konsistente Überschreitung der DRLs sollte eine formelle Überprüfung und Optimierung der lokalen Untersuchungsprotokolle auslösen. Diese Referenzwerte werden auf verschiedenen Ebenen etabliert, wobei europäische pädiatrische DRLs für Thoraxuntersuchungen nach Gewichtsklassen stratifiziert sind [12].

Die entscheidende Limitation traditioneller DRLs liegt jedoch in ihrer mangelnden Spezifität für die klinische Fragestellung. Ein Low-dose-CT des Thorax zur Metastasensuche bei einem onkologischen Patienten sollte naturgemäß eine deutlich niedrigere Strahlenexposition aufweisen als eine HRCT zur Abklärung einer potenziellen interstitiellen Lungenerkrankung beim selben Kind. Das EUCLID-Projekt hat diesen Bedarf erkannt und für Erwachsene indikationsbasierte DRLs eingeführt [43]. Für die Pädiatrie steht eine solch differenzierte Herangehensweise noch aus und ist dringend erforderlich.

Eine wegweisende Weiterentwicklung kommt aus den USA, wo das American College of Radiology (ACR) 2023 basierend auf den Daten seines Dose Index Registry (DIR) neue pädiatrische Referenzwerte publiziert hat. Dieser innovative duale Ansatz definiert zwei Niveaus:

- Diagnostic Reference Levels (DRLs): Weiterhin basierend auf dem 75. Perzentil, dienen sie als Obergrenze, die zur Reflektion und Überprüfung anregen soll.
- Achievable Doses (ADs): Diese basieren auf dem 50. Perzentil (Median) und stellen ein anspruchsvolleres Ziel für die Dosisoptimierung dar. Sie repräsentieren die Dosiswerte, die von gut optimierten Abteilungen routinemäßig erreicht werden.

Dieses System etabliert somit nicht nur eine *rote Linie*, sondern auch ein klares *Best-Practice-Ziel* und fördert so proaktiv die kontinuierliche Qualitätsverbesserung in der pädiatrischen Bildgebung [19].

### Der „Halbe-Schichtdicke-Ansatz“ als praktischer Optimierungszyklus

**Abb. 5:**
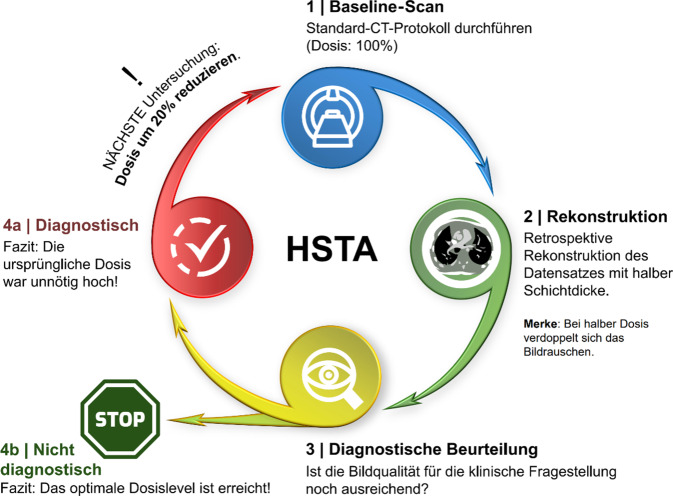
Halber-Schichtdicke-Ansatz („half slice thickness approach“, HST) zur schrittweisen Optimierung der Untersuchungsprotokolle in der Computertomographie. (Mod. nach [3, 31])

Wie oben dargelegt, besteht der erste Schritt zur Optimierung lokaler CT-Protokolle darin, die lokalen DRLs in einer Gesundheitseinrichtung zu definieren und sie mit nationalen oder europäischen DRLs zu vergleichen. Man sollte sich bewusst sein, dass niedrigere lokale DRLs im Vergleich zu nationalen oder europäischen DRLs nicht unbedingt die beste Optimierung bedeuten; eine weitere Anpassung der CT-Protokolle kann je nach Patientengröße, klinischer Fragestellung oder verfügbarer Ausrüstung erforderlich sein. Auf dieser Ebene kann der *Halbe-Schichtdicke-Ansatz* hilfreich sein [3]. Dieser kann ohne Phantome oder komplizierte Berechnungen durchgeführt werden. Als ersten Schritt rekonstruiert der Bediener einen Standard-CT-Scan mit halber Schichtdicke, und der Kinderradiologe bewertet die Bildqualität. Wenn die Bildqualität immer noch diagnostisch ist, sollte man dieses Strahlenniveau als 100 % Überschussdosis betrachten, und der nächste Scan sollte mit 20 % weniger Strahlung durchgeführt werden. Dieser Kreis kann wiederholt werden, bis die Bildqualität als nichtdiagnostisch angesehen wird, was bedeuten würde, dass das optimale Strahlenniveau erreicht wurde (Abb. 5).

## Photon-Counting-CT

Kürzlich wurde ein neuer Photon-Counting-Detektor (PCD) in die klinische Praxis eingeführt. Bei dieser Technologie werden Photonen von einer speziellen Halbleiterschicht ohne szintillierendes Material detektiert, was die Effektivität von CT-Detektoren erhöht und zu einer vielversprechenden Dosisreduktion führt. Dieses effektivere Detektormaterial ermöglicht eine verbesserte räumliche Auflösung mit weniger elektronischem Rauschen und verbessert die Detektion niederenergetischer Photonen, die die meisten Informationen für kontrastarme Darstellungen tragen [36].

Die PCD-CT stellt eine Innovation dar, deren zentrales Hardware-Upgrade in Detektoren mit kleineren Pixeln als bei Standarddetektoren und der direkten Umwandlung einzelner Röntgenstrahlen in elektrische Signale liegt [46]. Dies resultiert in CT-Bildern mit höherer räumlicher Auflösung (bis zu 0,2 mm) und reduziertem Gesamtrauschen, ohne dass eine höhere Strahlendosis erforderlich ist [16]. Diese Eigenschaften sind entscheidend für die pädiatrische Bildgebung, insbesondere bei Säuglingen und Kleinkindern, bei denen anatomische Strukturen deutlich kleiner sind als bei Erwachsenen und bei denen die Dosis so gering wie möglich gehalten werden muss [16]. Die PCD-CT hat das Potenzial, das Bildrauschen zu reduzieren, die räumliche und Kontrastauflösung zu verbessern und multispektrale Fähigkeiten bereitzustellen, alles bei einer möglichen Verringerung der Gesamtstrahlendosis [9]. Diese Effekte können genutzt werden, um die Dosis jodhaltiger Kontrastmittel zu reduzieren und möglicherweise Mehrphasenbilder durch eine einzige Akquisitionstechnik zu erhalten. Erste klinische Erfahrungen zeigen vielversprechende pädiatrische Anwendungen der PCD-CT in verschiedenen anatomischen Regionen [9, 16]. Die Technologie ist besonders vorteilhaft für Kinder, die wiederholte Scans benötigen, für sehr kleine Säuglinge zur Darstellung feiner anatomischer Details wie sie bei interstitiellen Lungenerkrankungen vorkommen können [9]. Die Fähigkeit, Photonenenergien zu unterscheiden, ermöglicht die inhärent spektrale Bildgebung. Obwohl die Erfahrungen bei pädiatrischen Patienten zunehmen und als sehr vielversprechend gelten, steht die breite Implementierung und weitere Forschung noch aus, um das volle Potenzial dieser Technologie für Kinder auszuschöpfen [9, 16].

## Ausblick

Die moderne pädiatrische Thorax-CT hat sich von einer rein morphologischen zu einer multiparametrischen Methode gewandelt. Der Wandel vom passiven Bildbetrachter zum aktiven Protokollmanager, der für jede Indikation das optimale Verhältnis von Bildqualität und Dosis justiert, ist die zentrale Anforderung an den heutigen Kinderradiologen. Technologien wie die Photon-Counting-CT werden uns dabei unterstützen, diesem Anspruch auch in Zukunft noch besser gerecht zu werden.

## Fazit für die Praxis

- Indikation kritisch prüfen: Jede Computertomographie (CT) des Thorax bei einem Kind muss streng indiziert und gegen strahlenfreie Alternativen (Sonographie, Magnetresonanztomographie) abgewogen werden.
- Protokolle individualisieren: Passen Sie kV, mAs und Rekonstruktionsalgorithmus immer an die Größe des Kindes und die spezifische klinische Fragestellung an.
- Moderne Technik nutzen: Axialer Scanmodus und hoher Pitch zur Vermeidung von Sedierung, Niedrig-kV-Technik für CT-Angiographie (CTA) und iterative/Deep-Learning-Rekonstruktion sind Standard.
- In 3D denken: Maximum-Intensitäts-Projektion (MIP) zur Nodulisuche, Minimum-Intensitäts-Projektion (MinIP) zur Atemwegsbeurteilung und Volumen-Rendering-Technik (VRT) zur präoperativen Planung sind integraler Bestandteil der Diagnostik.
- Dosis überwachen und optimieren: Vergleichen Sie Ihre lokalen Dosiswerte regelmäßig mit Benchmarks (diagnostische Referenzwerte [DRLs] und Achievable Doses [ADs]) und nutzen Sie pragmatische Methoden zur kontinuierlichen Verbesserung.
